# Expanding horizons in overcoming therapeutic resistance in castration-resistant prostate cancer: targeting the androgen receptor-regulated tumor immune microenvironment

**DOI:** 10.20892/j.issn.2095-3941.2023.0256

**Published:** 2023-08-28

**Authors:** Bisheng Cheng, Hai Huang

**Affiliations:** Department of Urology, Sun Yat-sen Memorial Hospital, Sun Yat-sen University, Guangzhou 510120, China

Castration-resistant prostate cancer (CRPC) poses a major treatment challenge, because disease progression occurs despite androgen deprivation therapy (ADT)^1^. Overcoming therapeutic resistance in CRPC remains a critical unmet need in clinical practice. The androgen receptor (AR) signaling pathway plays a critical role in prostate cancer development and progression, and is also involved in regulating the tumor immune microenvironment (TIME). The TIME is a complex network of immune cells, stromal cells, and soluble factors that interact with tumor cells and influence tumor growth, invasion, and response to therapy. Recent studies have highlighted the effects of AR signaling on immune cell function and immune-mediated anti-tumor responses within the tumor microenvironment^2,3^. Androgens modulate the expression of immune checkpoint molecules, cytokines, and chemokines, thereby shaping the immune landscape of tumors^4,5^. Targeting the TIME component regulated by the AR is a promising strategy to overcome therapeutic resistance in CRPC. By understanding the interplay between AR signaling and immune cell function, innovative therapeutic approaches may be developed that enhance anti-tumor immune responses, overcome immune evasion mechanisms, and sensitize CRPC to existing therapies.

## The AR signaling axis in CRPC and clinical dilemma

**Figure 1:**
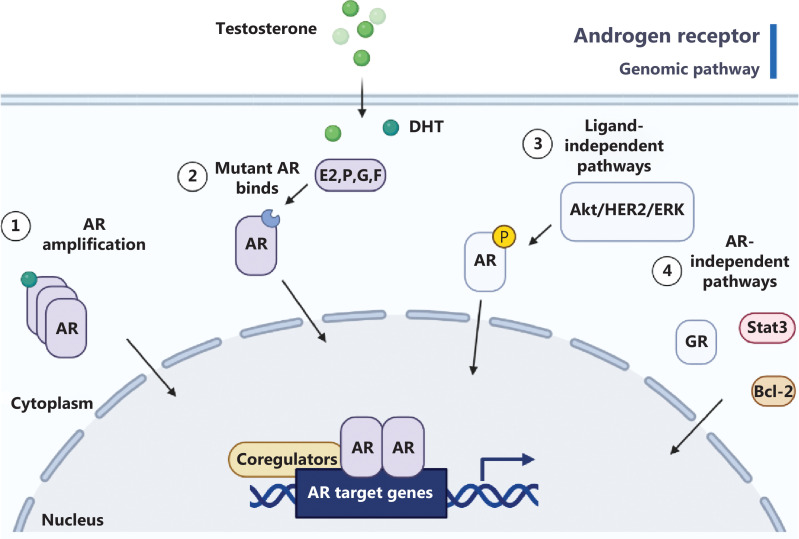
Illustration of the androgen receptor (AR) genomic pathway. ① Elevated AR expression combined with continuous production of steroids by the tumor. ② Non-specific binding and activation of mutant AR by alternative ligands, including estrogen (E2), progesterone (P), glucocorticoids (G), and flutamide (F). ③ Ligand-independent mechanisms of AR activation involve the Akt, HER2, and Ack1 kinases, which lead to AR phosphorylation, as well as long non-coding RNAs (e.g., PCGEM1), which bind AR and enhance the transcription of genes targeted by AR. ④ AR-independent pathways contributing to cancer cell survival and growth, mediated by Stat3 signaling or upregulation of anti-apoptotic Bcl-2. The glucocorticoid receptor (GR) activates a similar set of AR target genes critical for cancer cell survival.

The AR plays a critical role in prostate cancer progression by regulating genes involved in cell growth, survival, and differentiation. In CRPC, AR signaling remains active despite androgen depletion through ADT. Ligand-independent activation is a mechanism contributing to AR activation in CRPC, wherein alternative signaling pathways bypass the need for androgens^4–6^. Growth factor receptors (e.g., EGFR and IGF-1R) and intracellular signaling cascades (e.g., PI3K/Akt and MAPK) activate AR signaling without androgens. AR gene amplification is another mechanism associated with elevated AR expression, aggressiveness, and resistance to hormonal therapies in CRPC^7^. AR splice variants, particularly AR-V7, lacking the ligand-binding domain, contribute to therapeutic resistance (**Figure 1**). The role of the 4EBP-eIF4E axis in AR-deficient prostate cancer presents intriguing opportunities for therapeutic intervention. Recent findings have highlighted translation initiation as a critical driver in AR-deficient prostate cancer, wherein the AR negatively regulates protein synthesis through transcriptional control of 4EBP1^8^. Understanding these mechanisms is crucial for developing targeted therapies to inhibit AR signaling and overcome resistance in CRPC. Strategies include novel AR antagonists, inhibitors of AR signaling pathways, and combination therapies targeting multiple components of the AR signaling axis. Overcoming persistent AR signaling in CRPC is a major clinical challenge requiring elucidation of these activation mechanisms.

## Unraveling the TIME in CRPC

The AR signaling axis drives prostate cancer cell growth and survival. However, emerging research suggests that AR signaling also influences the composition and function of immune cells within the TIME. Understanding the intricate interplay between AR signaling and the immune microenvironment is essential for developing effective therapeutic approaches^9,10^. One key aspect of the AR-regulated TIME is the modulation of immune checkpoint molecules. Immune checkpoints, such as programmed cell death protein 1 (PD-1), programmed death-ligand 1 (PD-L1), and cytotoxic T-lymphocyte-associated protein 4 (CTLA-4), play crucial roles in maintaining immune homeostasis. In CRPC, the upregulation of immune checkpoint molecules, including B7-H3, contributes to immune evasion and resistance to immune-based therapies^11,12^. Consequently, targeting these checkpoint molecules with immune checkpoint inhibitors has shown promising results in clinical trials, thereby highlighting the value of immune modulation in CRPC treatment. Cytokines and chemokines are also important components of the AR-regulated TIME. The cytokines interleukin-6 (IL-6) and tumor necrosis factor-alpha (TNF-α) promote inflammation and tumor progression in CRPC^13,14^. These cytokines stimulate the production of chemokines and growth factors, which in turn regulate the recruitment and activation of immune cells, including tumor-infiltrating lymphocytes and myeloid-derived suppressor cells (MDSCs). The presence of specific chemokines within the TIME, such as CCL2 and CXCL12, influences immune cell trafficking and the overall immune response. Therefore, targeting these cytokines and chemokines holds promise for modulating the immune microenvironment in CRPC^15,16^. Tumor cells use various immunosuppressive mechanisms to evade immune surveillance in CRPC. Regulatory T cells play a major role in immune tolerance by suppressing the activity of effector immune cells. Their presence in the TIME dampens the antitumor immune response, thus promoting tumor growth and progression. Additionally, MDSCs contribute to immunosuppression within the TIME. MDSCs inhibit T cell activation and function, and promote angiogenesis and tumor metastasis. Targeting these immunosuppressive cell populations and pathways may promisingly enhance the efficacy of immunotherapeutic approaches in CRPC. In conclusion, the AR-regulated TIME plays a crucial role in shaping the immune response in CRPC. Immune checkpoint molecules, cytokines, and chemokines are critical in modulating the immune microenvironment, whereas tumor cells use various immunosuppressive mechanisms to evade immune surveillance. Targeting the AR-regulated TIME is a promising avenue for developing novel therapeutic targets and effective immunotherapeutic strategies in the treatment of CRPC. A comprehensive understanding of the intricate interactions within the TIME will be essential for advancing knowledge and improving the outcomes of patients with CRPC.

## Crosstalk between AR signaling and immune cells within the TIME

**Figure 2:**
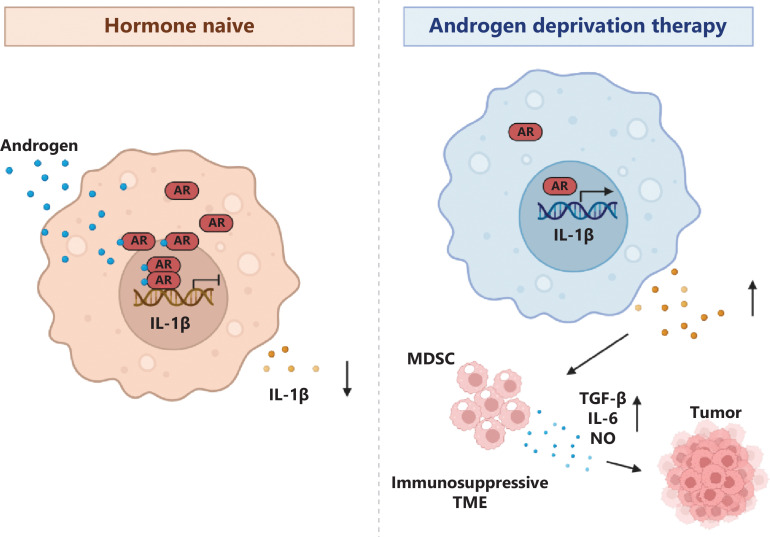
Illustration of AR-mediated repression of IL-1β and effects on the TME. The figure illustrates the role of AR as a transcriptional repressor of IL-1β in tumor-associated macrophages (TAMs). In the absence of androgen deprivation therapy (ADT), AR restrains the expression and secretion of IL-1β in TAMs, thereby maintaining immune homeostasis within the tumor microenvironment (TME). After ADT, AR’s inhibition of IL-1β is alleviated, thus resulting in excessive expression and secretion of IL-1β in TAMs, and establishing an immune suppressive microenvironment.

**Figure 3:**
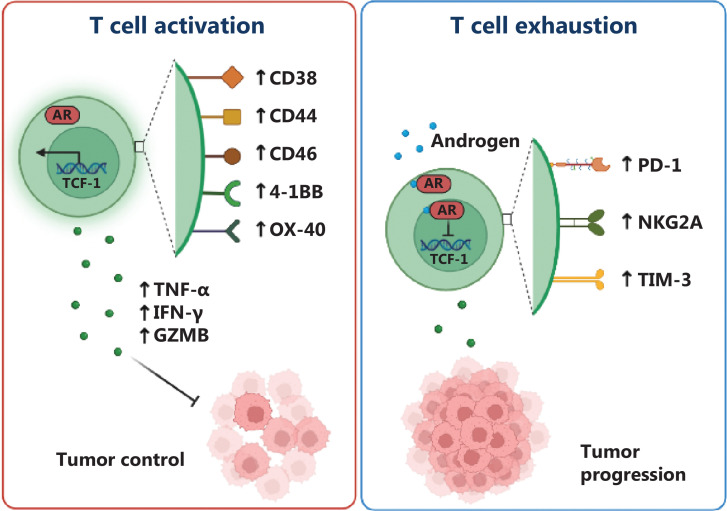
Illustration of AR regulation of tumor-infiltrating CD8+ T cell activity and stemness. The AR inhibits the activity of male tumor-infiltrating CD8+ T cells, thus resulting in decreased effector function and impaired cytotoxicity against tumor cells. In addition, the AR negatively affects the stemness of CD8+ T cells, thereby decreasing self-renewal capability and compromising long-term persistence within the tumor microenvironment.

Emerging evidence suggests complex and bidirectional crosstalk between AR signaling and immune cells within the TIME. AR signaling has been found to modulate immune cell recruitment, activation, and differentiation within the TIME. Activation of the AR pathway promotes the secretion of chemokines and cytokines by tumor cells, which in turn attract immune cells to tumor sites. Moreover, AR activation affects the expression of adhesion molecules on endothelial cells, thus facilitating the infiltration of immune cells into the tumor microenvironment. These findings highlight the role of AR signaling in shaping immune cell composition within the TIME. Additionally, AR signaling influences immune cell function in CRPC. ADT inhibits AR signaling and decreases androgen levels. This androgen depletion has been shown to affect immune cell populations and functions. For instance, ADT increases the number and activity of natural killer cells, and decreases MDSCs. Furthermore, ADT has been associated with overcoming the inhibitory effects of AR on IL-1β, thus leading to excessive expression and secretion of IL-1β in TAMs. IL-1β induces MDSC accumulation, thereby inhibiting the activation of cytotoxic T cells and leading to an immune suppressive microenvironment (**Figure 2**)^4^. Moreover, androgen signaling suppresses T-cell immunity against cancer in males by upregulating the expression of USP18, and consequently inhibiting TAK1 phosphorylation and subsequent activation of NF-κB in antitumor T cells. Decreasing testosterone synthesis through surgical castration or administration of the small-molecular inhibitor abiraterone significantly enhances the antitumor activity of T cells and increases the efficacy of anti-PD-1 immunotherapy (**Figure 3**)^17^. Moreover, the crosstalk between AR signaling and immune cells within the tumor microenvironment extends to the involvement of the MNK1/2-eIF4E axis, which plays a crucial role in regulating mRNA translation of pro-proliferative and anti-apoptotic genes in cancer. By integrating signals from both oncogenic and immune signaling pathways, MNK1/2 modulates the function of immune cells within the TIME, thus downregulating of multiple immune suppressive proteins, including PD-1, PD-L1, and IL-10^18^. Notably, ongoing investigations of the MNK1/2 inhibitor eFT508 in CRPC are providing valuable insights into its potential as a therapeutic strategy. These observations highlight the effects of AR signaling on immune cell function and potential implications for therapeutic strategies targeting the AR-regulated TIME. The bidirectional crosstalk between AR signaling and immune cells in the TIME enables opportunities for combination therapies that target both the AR-mediated pathway and the immune system. Dual-targeting approaches, such as combining AR pathway inhibitors with immune checkpoint inhibitors, have shown promising results in preclinical and clinical studies. These combinatorial strategies are aimed at disrupting the immunosuppressive effects of AR signaling while unleashing the full potential of the immune system to mount an effective anti-tumor response. In conclusion, bidirectional crosstalk exists between AR signaling and immune cells within the TIME in CRPC. AR signaling influences immune cell recruitment, activation, and function, whereas immune cells in turn influence AR signaling through various mechanisms. Understanding the molecular intricacies of this crosstalk will be crucial for developing effective therapeutic strategies targeting both the AR-regulated TIME and the immune system in CRPC.

## Personalized immunotherapeutic strategies in CRPC

The advent of personalized medicine has opened new avenues for the treatment of CRPC. This section focuses on the potential of genomic and immune profiling in CRPC to identify predictive biomarkers that can guide patient stratification and treatment selection. Through understanding the individual characteristics of each patient’s tumors, personalized immunotherapeutic strategies can be developed to enhance treatment efficacy and overcome therapeutic resistance. Genomic profiling is crucial in identifying the genetic alterations and molecular subtypes associated with treatment response and prognosis in CRPC. Recent advances in next-generation sequencing technologies have enabled the identification of somatic mutations, copy number alterations, and gene fusions in prostate cancer genomes. These genomic alterations can serve as potential predictive biomarkers for immunotherapy response. For example, tumors with DNA repair defects, such as BRCA1/2 mutations, have elevated sensitivity to immune checkpoint inhibitors^19^. Moreover, immune profiling of the tumor microenvironment can provide valuable insights into the immune response landscape and help identify immune-associated biomarkers that predict response to immunotherapy. Integrating immunotherapeutic strategies with existing treatments holds promise in enhancing treatment outcomes in CRPC. Approaches that combine immune checkpoint inhibitors with ADT or chemotherapy have shown encouraging results. ADT modulates the immune microenvironment by altering androgen levels, thereby sensitizing tumors to immune checkpoint inhibitors. Additionally, chemotherapy-induced tumor cell death releases tumor antigens and promotes immune activation, thus making it attractive in immunotherapy applications. The synergistic effects of these combinations may potentially overcome therapeutic resistance and improve patient outcomes. Another important aspect of personalized immunotherapeutic strategies is the consideration of individual heterogeneity in CRPC. Each patient’s tumor may contain unique genomic alterations and immunological characteristics, thus necessitating a tailored approach to treatment. Precision medicine approaches, such as identifying neoantigens specific to an individual’s tumor, can guide the development of personalized vaccines or adoptive cell therapies. Additionally, monitoring dynamic changes in the tumor microenvironment during treatment can help guide therapeutic decisions and optimize treatment strategies. In conclusion, personalized immunotherapeutic strategies have the potential to revolutionize the treatment of CRPC. Genomic and immune profiling can aid in the identification of predictive biomarkers, thus allowing for patient stratification and selection of appropriate immunotherapeutic approaches. Integration with existing treatments and consideration of individual tumor heterogeneity are essential for maximizing treatment efficacy and overcoming therapeutic resistance. Continued research and clinical trials are needed to refine and validate personalized immunotherapeutic strategies in CRPC.

## Future perspectives and conclusion

The expanding prospects for targeting the AR-regulated TIME hold great promise for overcoming therapeutic resistance in CRPC. One key future area of focus will be developing combination therapies that target both the AR signaling axis or its downstream molecules, and the immune microenvironment. Concurrent inhibition of AR signaling within TIME components and enhancement of the anti-tumor immune response can achieve synergistic effects that ultimately improve treatment outcomes. The identification of optimal combination regimens and treatment schedules will be crucial for maximizing efficacy while minimizing toxicity. Additionally, the development of novel immunomodulatory agents specifically designed to target the AR-regulated TIME is an exciting area of research. These agents selectively modulate immune cell function and enhance anti-tumor activity within the TIME, thereby overcoming tumor cells’ immunosuppressive mechanisms. Strategies such as the development of immune cell-targeted therapies, cytokine-based therapies, and novel immune checkpoint inhibitors may revolutionize the treatment landscape of CRPC.

Furthermore, the integration of artificial intelligence (AI) and machine learning (ML) approaches will be critical in unraveling the complexities of the AR-regulated TIME. AI and ML algorithms can analyze large-scale genomic and proteomic data, identify patterns, and predict treatment responses and outcomes. These integrated methods may facilitate the discovery of novel biomarkers, aid in patient stratification, and guide treatment decision-making, thus ultimately leading to personalized and precise therapeutic approaches. In conclusion, the expanding horizons in targeting the AR-regulated TIME offer promising prospects for overcoming therapeutic resistance in CRPC. Future research directions include the development of combination therapies and novel immunomodulatory agents, and the integration of AI and ML in unraveling the complexities of the AR-regulated TIME. Leveraging these advancements should support the optimization of treatment strategies and improvement in patient outcomes, and ultimately enable therapeutic resistance in CRPC to be overcome.

## References

[1] Gillessen S, Bossi A, Davis ID, de Bono J, Fizazi K, James ND (2023). Management of patients with advanced prostate cancer. Part I: Intermediate-/High-risk and locally advanced disease, biochemical relapse, and side effects of hormonal treatment: report of the advanced prostate cancer consensus conference 2022. Eur Urol.

[2] Wang H, Li N, Liu Q, Guo J, Pan Q, Cheng B (2023). Antiandrogen treatment induces stromal cell reprogramming to promote castration resistance in prostate cancer. Cancer Cell.

[3] Li N, Liu Q, Han Y, Pei S, Cheng B, Xu J (2022). ARID1A loss induces polymorphonuclear myeloid-derived suppressor cell chemotaxis and promotes prostate cancer progression. Nat Commun.

[4] Wang D, Cheng C, Chen X, Wang J, Liu K, Jing N (2023). IL-1beta is an Androgen-Responsive target in macrophages for immunotherapy of prostate cancer. Adv Sci (Weinh).

[5] Toniutto P, Shalaby S, Mameli L, Morisco F, Gambato M, Cossiga V (2023). Role of sex in liver tumor occurrence and clinical outcomes: a comprehensive review. Hepatology.

[6] Wang Q, Li Z, Yang J, Peng S, Zhou Q, Yao K (2021). Loss of NEIL3 activates radiotherapy resistance in the progression of prostate cancer. Cancer Biol Med.

[7] Del RM, Biasco E, Crucitta S, Derosa L, Rofi E, Orlandini C (2017). The detection of androgen receptor splice variant 7 in plasma-derived exosomal RNA strongly predicts resistance to hormonal therapy in metastatic prostate cancer patients. Eur Urol.

[8] Liu Y, Horn JL, Banda K, Goodman AZ, Lim Y, Jana S (2019). The androgen receptor regulates a druggable translational regulon in advanced prostate cancer. Sci Transl Med.

[9] Cheng B, Tang C, Xie J, Zhou Q, Luo T, Wang Q (2023). Cuproptosis illustrates tumor micro-environment features and predicts prostate cancer therapeutic sensitivity and prognosis. Life Sci.

[10] Hirz T, Mei S, Sarkar H, Kfoury Y, Wu S, Verhoeven BM (2023). Dissecting the immune suppressive human prostate tumor microenvironment via integrated single-cell and spatial transcriptomic analyses. Nat Commun.

[11] Shi W, Wang Y, Zhao Y, Kim JJ, Li H, Meng C (2023). Immune checkpoint B7-H3 is a therapeutic vulnerability in prostate cancer harboring PTEN and TP53 deficiencies. Sci Transl Med.

[12] Sridaran D, Chouhan S, Mahajan K, Renganathan A, Weimholt C, Bhagwat S (2022). Inhibiting ACK1-mediated phosphorylation of C-terminal Src kinase counteracts prostate cancer immune checkpoint blockade resistance. Nat Commun.

[13] Patel R, Fleming J, Mui E, Loveridge C, Repiscak P, Blomme A (2018). Sprouty2 loss-induced IL6 drives castration-resistant prostate cancer through scavenger receptor B1. EMBO Mol Med.

[14] Sharma J, Gray KP, Harshman LC, Evan C, Nakabayashi M, Fichorova R (2014). Elevated IL-8, TNF-alpha, and MCP-1 in men with metastatic prostate cancer starting androgen-deprivation therapy (ADT) are associated with shorter time to castration-resistance and overall survival. Prostate.

[15] Jung Y, Cackowski FC, Yumoto K, Decker AM, Wang J, Kim JK (2018). CXCL12gamma promotes metastatic castration-resistant prostate cancer by inducing cancer stem cell and neuroendocrine phenotypes. Cancer Res.

[16] Lee GT, Kwon SJ, Kim J, Kwon YS, Lee N, Hong JH (2018). WNT5A induces castration-resistant prostate cancer via CCL2 and tumour-infiltrating macrophages. Br J Cancer.

[17] Zhang X, Cheng L, Gao C, Chen J, Liao S, Zheng Y (2023). Androgen signaling contributes to sex differences in cancer by inhibiting NF-kappaB activation in T cells and suppressing antitumor immunity. Cancer Res.

[18] Xu Y, Poggio M, Jin HY, Shi Z, Forester CM, Wang Y (2019). Translation control of the immune checkpoint in cancer and its therapeutic targeting. Nat Med.

[19] Barata P, Agarwal N, Nussenzveig R, Gerendash B, Jaeger E, Hatton W (2020). Clinical activity of pembrolizumab in metastatic prostate cancer with microsatellite instability high (MSI-H) detected by circulating tumor DNA. J Immunother Cancer.

